# Lack of implementation of Hepatitis B Virus (HBV) vaccination policy in household contacts of HBV carriers in Italy

**DOI:** 10.1186/1471-2334-9-86

**Published:** 2009-06-07

**Authors:** Paola Scognamiglio, Enrico Girardi, Mario Fusco, Pierluca Piselli, Silvana Russo Spena, Carmela Maione, Francesco Aurelio Pisanti, Diego Serraino

**Affiliations:** 1 Department of Epidemiology and Preclinical Research, Istituto Nazionale per le Malattie Infettive, "L. Spallanzani" IRCCS, Via Portuense 292, 00149, Rome, Italy; 2 Registro Tumori della Regione Campania, ASL NA4 Brusciano, Naples, Italy; 3 Unit of Epidemiology and Prevention, ASL NA4 Brusciano, Naples, Italy; 4 Clinical Pathology Unit, ASL NA4 Brusciano, Naples, Italy; 5 Unit of Epidemiology and Biostatistics, Centro di Riferimento Oncologico IRCCS, Aviano, Pordenone, Italy; 6 Members of the Collaborating Study Group are cited in the acknowledgements section

## Abstract

**Background:**

In Italy, HBV vaccination is recommended and offered free of charge through the National Health Service to selected population groups – e.g., family members of an HBsAg carrier, healthcare workers, newborns and those who were 12-years old in 1991. However, a significant proportion of cases of acute hepatitis B still occur in Italy among persons who should have been vaccinated. We analysed HBV sero-prevalence data of two vaccination target populations (people born after 1980 and household contacts of an HBV carrier) living in a southern Italian area in order to evaluate HBV vaccine coverage and its possible determinants.

**Methods:**

Between 2003 and 2006, we carried out a cross-sectional, population-based, sero-epidemiological survey on HBV infection on 4496 randomly selected individuals (aged 20 years or more) from the general population of the province of Naples. Sera were tested for antibodies to hepatitis B core antigen (anti-HBc) and to hepatitis B surface antigen (anti-HBsAg) by commercial immunoassays. Prevalence of past or current HBV infection and of HBV vaccination-induced immunity was calculated in two vaccination target populations. To analyze the association of epidemiological and socioeconomic characteristics with HBV vaccination of household contacts, we calculated crude and multiple logistic regression (MLR) odds ratio (OR).

**Results:**

Prevalence of HBV vaccine-induced immunity (anti-HBs alone) was much lower among household contacts (25%) than among those who had been targeted for universal adolescent vaccination (81.6%). Male sex, older age, unemployment and lower education levels were associated to lower immunization rates.

**Conclusion:**

Understanding the different uptake of hepatitis B vaccination in these populations may provide useful information for optimizing vaccination campaigns in other contexts. Our data clearly demonstrated the need of improving the uptake of vaccination for household contacts of HBV carriers.

## Background

In Italy, since 1984 HBV vaccination has been recommended and offered free of charge by the National Health Service to high-risk groups – e.g., family members of HBsAg carriers, healthcare workers. In 1991, vaccination became mandatory for all newborns and for twelve year-old adolescents [1]. According to the Acute Hepatitis National Surveillance System (SEIEVA), the incidence of acute hepatitis B decreased from 5.1 cases per 100,000 persons in 1991 to 1.3 per 100,000 persons in 2005. However, a significant proportion of cases of acute hepatitis B still occur in Italy among persons who should have been vaccinated [1].

To provide further insight into missed opportunities for HBV prevention in Italy, we analyzed HBV sero-prevalence data of two target populations for HBV vaccination, i.e., people born after 1980 and household contacts of an HBV carrier, collected in a population-based survey on viral hepatitis infections [2]. The survey was conducted in the province of Naples, an area of southern Italy with the highest incidence rates of liver cancer in Europe and where about 90% of liver cancers are attributable to infection with hepatitis C virus (HCV) and/or HBV [3].

Aim of this analysis was to compare HBV vaccination coverage between two target populations.

## Methods

Between 2003 and 2006, we carried out a cross-sectional, population-based, sero-epidemiological survey on hepatitis virus infections in the province of Naples. Methods of this survey have been previously described in detail [2]. Briefly, 4496 randomly selected individuals (aged 20 years or more), after signing informed consent, donated a blood sample and completed a standardized questionnaire, which included questions on HBV infected households and on HBV vaccination. The study protocol conformed to the 1975 Declaration of Helsinki and had been approved by the CCR Board of Ethics.

Sera were tested for hepatitis B surface antigen (HBsAg) and for antibodies to HBV-core antigen (anti-HBc) using enzyme immunoassays (Cobas Core ll, Roche Diagnostics, Indianapolis, IN, USA); for antibodies to HBV-surface antigen (anti-HBs) by enzyme immunoassays (Anti-HBs Quant EIA II – Roche Diagnostics): samples with a reactivity >10 IU/L were considered positive [4].

For the purpose of this analysis, positive results for anti-HBc were considered to indicate past or current HBV infection, while positivity for anti-HBsAg (anti-HBs) only was considered indicative of HBV vaccination-induced immunity. Individuals who tested negative for all HBV serological markers were considered as non immune/non infected.

### Statistical analysis

Prevalence of past or current HBV infection and of HBV vaccination-induced immunity was calculated in two vaccination target populations i.e. persons born ≥ 1980 and household contacts of an HBV carrier. To analyze the association of epidemiological and socioeconomic characteristics with HBV vaccination of household contacts, we compared, among those born before 1980, individuals with immune protection with those not immune/not infected. As a measure of association, we calculated odds ratio (OR) and multivariate logistic regression odds ratio (MLR-OR) and their 95% confidence intervals (95% CI). The MLR was adjusted for gender, age, as a priori chosen variables, and for variables significantly associated with immune protection in univariate analysis. We also performed a sensitivity analysis considering as "vaccinated", subjects that reported to have been vaccinated but testing negative for anti-HBs.

To evaluate agreement between vaccination status according to self-report and that obtained from laboratory results, we calculated the percentage of agreement and the kappa (k) statistic. Statistical analyses were performed using SPSS package (version 15.00 SPSS Inc., Chicago, Illinois).

## Results

Among 577 study subjects born between 1980 and 1983 who should have been vaccinated at the age of 12 years, the overall prevalence of past or current HBV infection was 3.3%. The prevalence of HBV vaccination-induced immunity was 81.6% (471/577).

Among 247 individuals born before 1980 who reported living with an HBV carrier, the rate of anti-HBs seropositivity was 15.4% (38/247). Excluding 95 subjects with past or current HBV infection, the prevalence of anti-HBs seropositivity among households was 25% (38/152). This proportion was significantly lower than that recorded among those born between 1980 and 1983 (p = 0.0000). The overall agreement among these individuals between self-reported and serologic evidence of vaccination was 87.2%, with a k = 0.58 (*p *= 0.0000).

The prevalence of HBV vaccination-induced immunity among not infected household contacts born before 1980 according to socio-demographic characteristics is presented in table 1. In the final multiple logistic regression model (including terms for gender, age, education and occupation) socio-economic factors significantly associated with HBV vaccination were female gender, younger age and being employed. Sensitivity analysis confirmed these associations (data not shown).

**Table 1:** HB vaccination-induced immunity among not infected households born before 1980 according to socio-demographic characteristics

	Vaccine-induced immunity^a^			
	**Not Vaccinated^b ^N (%)**	**Vaccinated^c ^N (%)**	**OR (95% CI)**	**MLR-OR^d ^(95% CI)**

Total	114 (75.0)	38 (25.0)		

***Gender***				
Male	47 (81.0)	11 (19.0)	1	1
Female	67 (71.3)	27 (28.7)	1.7 (0.8–3.8)	2.6 (1.0–6.6)

***Age***				
for each 10 year increase			0.42 (0.40–0.45)	0.60 (0.38–0.87)

***Education***				
0–8 years	74 (81.3)	17 (18.7)	1	1
≥ 9 years	40 (65.6)	21 (34.4)	2.3 (1.1–4.8)	1.4 (0.6–3.3)

***Current employment***				
Employed	40 (67.8)	19 (32.2)	1	1
Unemployed	10 (83.3)	2 (16.7)	0.4 (0.1–2.1)	0.2 (0.0–0.9)
Student, retired, housewife	55 (84.6)	10 (15.4)	0.4 (0.2–0.9)	0.4 (0.1–1.0)
Unknown	9 (56.2)	7 (43.8)	1.6 (0.5–5.1)	1.3 (0.4–4.4)

***Marital status***				
Ever Married	95 (77.2)	28 (22.8)	1	
Single	14 (63.6)	8 (36.4)	1.9 (0.7–5.1)	
Unknown	5 (71.4)	2 (28.6)	1.4 (0.2–7.4)	

***Infected family member***				
Partner	24 (80.0)	6 (20.0)	1	
Parent	26 (63.4)	15 (36.6)	2.3 (0.8–6.9)	
Other	34 (75.6)	11 (24.4)	1.3 (0.4–4.0)	
Unknown	30 (83.3)	6 (16.7)	0.8 (0.2–2.8)	

^a ^In some items, the sum does not add up to the total because of missing values.

^b ^All markers negative (HBsAg; anti-HBc; anti-HBs)

^c ^Only anti-HBs positive

^d ^Adjusted for gender, age and education and current employment

## Discussion

In our study, HBV vaccination coverage significantly differed between two target populations studied in the same geographical area at high HBV prevalence. Vaccine coverage among individuals who should have been vaccinated at the age of 12, in agreement with previous surveys [5,6], was quite high (81.6%) although lower than 93.6% coverage measured globally in Italy [6].

In contrast, the prevalence of anti-HBs seropositivity among household contacts of an HBV carrier was 15.4%, reaching 25% if we exclude those already infected, and we found that 46.1% of contacts were still HBV susceptible. Our data are similar to those found in a study in the United Kingdom showing that only 27% of susceptible contacts were immunized [7]. Understanding the different uptake of HBV vaccination in these two populations may provide useful information for optimizing HBV vaccination campaigns in other contexts.

In vaccination campaign for 12 year-old individuals, vaccine candidates were directly contacted by health districts vaccination units, after identification through lists of residents or lists of school attendees. Moreover a vaccination certificate was required for eighth grade final examination [5]. In contrast, vaccination for household contacts relies on referral to vaccination units of candidates by family physicians or by other health care providers involved in the diagnosis and care of HBV infection. Indeed, there is evidence of a low level of attention on family spread of HBV infection both by many patients and their physicians [8,9]. We could then speculate that active involvement of vaccination units may have been a key factor in promoting access to HBV vaccination. Our study also shows that individuals' factors may contribute to lack of immunization among household contacts of an HBV carrier. In fact in our study population, older persons, those unemployed and those with lower education were less likely to have HBV vaccination-induced immunity. These observations suggest that targeted strategies should be implemented to reach these persons.

We identified some potential limitations and weaknesses of our study. Considering anti-HBs a proxy of vaccination can introduce a misclassification of outcome because in some HBV vaccinated individuals antibody titres decline with time after the first vaccination, to below the level of 10 IU/L and some are non-responders [4]. On the other hand there was a good agreement between serological results and self reported data on vaccination, similar associations were found when we used self-reported vaccination as an outcome in the analysis instead of anti-HBs positivity. Another limitation of this study is that we have defined "household contacts of an HBV carrier" on the basis of a self-administered questionnaire but we do not have any clinical confirmation of this information.

## Conclusion

In conclusion, our data clearly demonstrated the need of improving the uptake of vaccination for household contacts of HBV carriers. Active recruitment of potential candidates into vaccination programs may be needed to achieve this goal. In this context information campaigns for HBV infected patients and health care providers may be needed and implementation of centralised, hospital-based immunisation programs, including home visits, should be considered [10].

## Competing interests

The authors declare that they have no competing interests.

## Authors' contributions

PS participated in the design of the study and drafted the manuscript. EG wrote the study design and drafted the manuscript. MF: coordinated the study procedures, particularly blood collection, in the area. PP participated in the design of the study and performed the statistical analysis. SRS and CM were responsible of data collection and quality control. FAP was responsible of blood testing in the study area. DS wrote the study design and collaborated in the interpretation of study findings. All authors have read and approved the final manuscript.

## Pre-publication history

The pre-publication history for this paper can be accessed here:

http://www.biomedcentral.com/1471-2334/9/86/prepub
